# Targeting the Virus Capsid as a Tool to Fight RNA Viruses

**DOI:** 10.3390/v14020174

**Published:** 2022-01-18

**Authors:** Lucie Hozáková, Barbora Vokatá, Tomáš Ruml, Pavel Ulbrich

**Affiliations:** Department of Biochemistry and Microbiology, University of Chemistry and Technology Prague, Technická 3, 166 28 Prague, Czech Republic; ctvrtecl@vscht.cz (L.H.); vokataa@vscht.cz (B.V.); rumlt@vscht.cz (T.R.)

**Keywords:** antivirals, virus inhibitor, antiviral compounds, capsid assembly, assembly inhibitor, capsid binding, capsid targeting

## Abstract

Several strategies have been developed to fight viral infections, not only in humans but also in animals and plants. Some of them are based on the development of efficient vaccines, to target the virus by developed antibodies, others focus on finding antiviral compounds with activities that inhibit selected virus replication steps. Currently, there is an increasing number of antiviral drugs on the market; however, some have unpleasant side effects, are toxic to cells, or the viruses quickly develop resistance to them. As the current situation shows, the combination of multiple antiviral strategies or the combination of the use of various compounds within one strategy is very important. The most desirable are combinations of drugs that inhibit different steps in the virus life cycle. This is an important issue especially for RNA viruses, which replicate their genomes using error-prone RNA polymerases and rapidly develop mutants resistant to applied antiviral compounds. Here, we focus on compounds targeting viral structural capsid proteins, thereby inhibiting virus assembly or disassembly, virus binding to cellular receptors, or acting by inhibiting other virus replication mechanisms. This review is an update of existing papers on a similar topic, by focusing on the most recent advances in the rapidly evolving research of compounds targeting capsid proteins of RNA viruses.

## 1. Introduction

The recent coronavirus pandemic has shown that emerging viruses pose a constant threat that should keep scientists and pharmacists alert. In addition, the search for new antivirals even against currently well-controlled viruses is justified by the risk of the emergence of drug-resistant mutants that occur either spontaneously or are selected in the presence of drugs applied during long-term infections. For the latter reason, new drugs that inhibit viral processes other than the most often targeted replication of the viral genome and virus maturation, are particularly desirable. This applies especially to viruses such as HIV-1, which still cannot be eliminated from infected individuals. The patients are dependent on a lifelong treatment with a combination of drugs preferentially targeting different steps of the virus life cycle. This approach has significantly extended their life expectancy and quality. Despite this undeniable advantage, combination therapy reducing the risk of disease progression is associated with adverse side effects and is very expensive. Moreover, the sword of Damocles still hangs over our heads, i.e., the threat of the emergence of new mutants that may be resistant to currently effective drug combinations. One of the essential steps in the life cycle of any viral replication is the formation of new viral particles mediated by the interaction of viral capsid proteins. Inhibition of this process blocks the virus assembly, thereby inhibiting the virus replication. Alternatively, compounds with the ability to strongly stabilize assembled viral particles can be used as inhibitors of its disassembly, thus inhibiting the steps following the entry of virus particles into the host cell. Viral capsid proteins interact with various host cell factors or cellular receptors, thus the compounds targeting and binding capsid proteins can be used also for the inhibition of interactions essential for the virus life cycle. In this review, we summarize the most current knowledge about compounds that target and interact with viral capsid proteins or assembled viral capsids, thus inhibiting the virus at various stages of its life cycle. We focus on RNA viruses, with one exception, the hepatitis B virus, where replication depends on the error-prone formation of an RNA intermediate, which stimulates the search for new inhibitors capable of alleviating the problem of new mutants resistant to inhibitors.

Since RNA viruses use various low fidelity RNA polymerases, they exhibit a high mutation rate for their replication, leading to high variability of their genome [1]. The presence of these mutations allows the rapid generation of new variants of the virus, resulting not only in the virus being able to evade the immune system but also in the development of their drug resistance [2]. In addition to the lack of proofreading activity of the polymerase, a high mutation rate is associated with the magnitude of the virus replication. Another factor is the size of the virus population. The larger the number of viruses, the greater the possibility of drug-resistant forms of the virus. An important factor is also the virus fitness, i.e., the ability of a given genetic variant to reproduce among other variants. Although mutations causing resistance to a particular drug mainly do not affect the pathogenicity of the virus, they represent a major problem in the treatment of virus infections and justify the search for alternative drugs, preferably with new targets for combination therapy.

## 2. Approaches to Find New Antiviral Compounds

Some of the modern approaches in drug design are based either on in silico molecular modeling using known viral target structures or on interaction or activity assays in a high-throughput screening (HTS) format. HTS of biologically active substances (candidate compounds) is usually performed either by a target-based (biochemical assay) or a cell-based approach.

The targeted approach requires cloning and expression of gene products targeted by the intended drug. This process is governed by several rules, such as that the protein must be in its native form and, if relevant, an appropriate posttranslational modification must be ensured. High concentration, purity, and stability are also desirable. Even so, the activity of the test substance in vitro may not correspond to the real situation in the organism, where various cell factors and pharmacokinetic and pharmacodynamic parameters can affect the function of the tested substance. The advantage of the target approach is the possibility to extend the experiments to characterize the binding parameters and determine the inhibitory mechanism.

The cell-based assay is a suitable alternative screening method to the target-based one. There is no need for detailed knowledge of the molecular target structure and the screening aims to identify the inhibitory effect of tested compounds in the cellular environment. This screening also provides basic information on the acute cytotoxicity of potential drugs before proceeding to the next phase of testing [3,4]. The disadvantage of this model, which does not reflect pharmacodynamic parameters, is compensated by the incomparably lower price, speed, and the lack of ethical considerations associated with animal studies.

Finally, it should be noted that selecting a substance by the HTS is only indicative and its suitability for further research and testing needs to be verified [5]. In future, due to the huge number of newly identified substances by the HTS, an increased screening capacity will be required and new strategies have to be introduced to reduce costs and enhance the efficiency of finding a usable drug targets [6].

Except for the abovementioned HTS or in silico screening, repurposing of drugs previously approved for other clinical applications can also be a direct path to the design and finding of new viral inhibitors, with the advantage of a significant reduction of time and cost of subsequent clinical trials.

## 3. Retroviruses and Search for New Antiretrovirals

Retroviruses are one of the genera of enveloped RNA viruses causing serious, often life-threatening global infections. Despite enormous efforts to find new antiretrovirals or effective vaccines, there are still millions of people living with HIV and more than one million are newly infected each year. Although HAART therapy is on the market and widely used, new mutants of the virus resistant to some of the used antiretrovirals are rapidly emerging. Furthermore, the unpleasant side effects of the current therapy should be mentioned. Thus, the search for new compounds that inhibit retroviruses, especially HIV, is of high importance.

The retroviral capsid (CA) protein is the main structural protein that provides crucial interactions during the assembly of immature particles and cores of mature virions. In the field of retrovirus particle assembly or the use of virus capsid protein as a tool for the delivery of virus replication inhibitors, extreme research efforts have been made to find promising antiretrovirals. To facilitate the design of effective compounds, the high-resolution structures of HIV-1 or Mason–Pfizer monkey virus immature particles and mature cores were solved [7,8] and the overall aspects of HIV-1 assembly were thoroughly reviewed [9]. Also, the methods for high-throughput screening of inhibitors of assembly and uncoating are currently available [4,10,11].

### 3.1. Capsid-Targeted Retroviral Inhibition

Capsid-targeted viral inactivation (CTVI) is based on a fusion of viral capsid protein with selected effector protein inhibiting the virus. The effector molecule targets either viral nucleic acid, virus replication enzymes, or affects protein assembly and viral particle formation. Constructed CA-fusion proteins are expressed by the infected cells and incorporated into the viral particle during assembly [12]. For proper functioning, stable expression in transfected cells, incorporation of fusion recombinant proteins into the nascent virion, retention of effector molecule function, and zero cytotoxicity, must be assured. The CTVI method has been successfully applied not only to inhibit retroviruses as HIV-1 and Murine leukemia virus (MLV) [13] but also other viruses like Hepatitis B virus (HBV) [14] and Dengue virus [15]. The active effector molecules used were either an enzyme, most often nuclease (but also lipase and protease), or a single-chain antibody with zero cytotoxicity [12]. The desired activity of the effector molecule is the degradation of viral nucleic acid (DNA or RNA), inhibition of replication enzymes [16] or protein shell disruption [17]. The effector molecule is usually attached to the C-terminus of the capsid protein facing the inner side of the particle, thus positioning this active molecule in contact with the nucleic acid and viral enzymes [18].

Natsoulis et al. [13] employed this approach by using staphylococcal nuclease (SN) to inhibit MLV. The authors demonstrated that the enzymatic activity of nuclease in the fusion with capsid protein is retained and suppresses the ability of the virus to reproduce 30–100-fold compared to the wild type. The advantage of SN is that it cleaves both single- and double-stranded RNA and DNA, it is inactive in the cytoplasm and thus non-toxic to the host cells due to the low intracellular concentration of Ca^2+^ ions, which are required for the SN activity. Since the extracellular environment is Ca^2+^ rich the SN, present in the fusion protein co-packaged into the virus particle, is thus activated after the virion is released from the infected cell into the extracellular space [19]. Interestingly, only a few Ca^2+^ ions are sufficient to maintain SN function in virions. SN, according to [20], can be replaced by *E. coli* RNase HI. This enzyme recognizes ssDNA, dsRNA, and mainly a hybrid of RNA with DNA, which is an intermediate in retrovirus replication, but in contrast to SN, it degrades only the RNA strand in the RNA–DNA duplex [21]. RNase HI is also non-toxic to cells and has a potent antiretroviral effect as was proved on MLV [22].

Although the use of the two abovementioned effector molecules appears promising, there is still a demand for new ones. For example, SAMHD1, a restriction factor of SAM domain- and HD domain-containing protein 1, which has been reported to have RNase activity, can also have some potential to serve as an efficient molecule in retroviral RNA degradation [23]. The use of recently developed CRISPR-Cas proteins with RNA cleavage properties can also be promising for their future use as the effector molecules not only against retroviruses. Cas13 and its subtypes cut single-stranded RNA and therefore represent an interesting option to fight RNA viruses, as was shown for SARS-CoV-2 and Influenza virus, when Abbott et al. [24] developed a CRISPR-Cas13-based strategy, PAC-MAN (prophylactic antiviral CRISPR in human cells) for specific viral RNA degradation.

The CTVI approach is still at the level of in vitro testing. In vivo tests to rule out its possible undesirable side effects must follow. Also, there is no guarantee that the prepared inhibitory systems will not be attacked or adversely interfere with the immune system. Except for the low cytotoxicity, there is also a need for stable expression of these CA-fusion proteins and their efficient incorporation into virions, while maintaining the antiviral activity of the effector molecule [12].

The greatest remaining challenge for the use of CTVI in vivo is the efficient delivery of the fusion protein (its coding gene), into the appropriate cells. Until the suitable gene delivery system is approved, the CTVI system will not be usable for humans. However, in animal models, this therapy has already been tested. Hepatitis B virus-transgenic mice were treated with an adenoviral vector carrying fusion protein consisting of HBV core protein and a ribonuclease, human eosinophil-derived neurotoxin [25], or the transgenic grass carp with the gene for fusion reoviral capsid protein and SN integrated into its genome was constructed to avoid its reoviral infection [26].

### 3.2. Antiretrovirals Aimed at Capsid–Capsid Protein Interaction

Another approach for inhibiting retrovirus replication is based on the direct interaction of a selected therapeutic molecule with the CA protein. The binding of such molecule to the CA protein then negatively influences or completely abolishes the assembly of immature retroviral particle or mature core or the virion core disassembly. Since the retroviral CA protein consists of two distinct structural domains, N-terminal (CA-NTD) and C-terminal (CA-CTD), various antiretrovirals targeting the CA protein can be divided into two groups, CA-NTD- and CA-CTD-binding. Based on their structure and nature, they can also be grouped into peptide-based and non-peptide-based inhibitors.

#### 3.2.1. CA-NTD-Binding Peptide Antiretrovirals

**Ankyrin**

Natural ankyrins are proteins mediating protein–protein interactions that can function both extra and intracellularly. Based on the analysis of their sequence-structure relationships, consensus ankyrin motifs were identified. By using these motifs as rigid parts of artificial ankyrins and by amino acid randomization of the rest of ankyrin molecules, large libraries of artificial proteins, called DARPins (Designed Ankyrin-Repeat Proteins) were generated. An artificial ankyrin named Ank^GAG^1D4 (16.5 kDa) was identified by phage-display screening as a binding partner to fused matrix and capsid domains of HIV-1 Gag polyprotein. HIV-1 production was significantly reduced in Ank^GAG^1D4 in SupT1 cells stably expressing Ank^GAG^1D4 compared to control cells [27]. Crystal structure and molecular docking analysis indicated that Ank^GAG^1D4 interacts with the CA-NTD of HIV-1 CA [28].

#### 3.2.2. CA-CTD-Binding Peptide Antiretrovirals

**CAI**

CAI (capsid assembly inhibitor) is a dodecamer peptide (Table 1), which was identified by phage display screening of CA binding proteins [29,30]. CA-CTD of HIV-1 CA protein is organized as a flattened four-helix bundle. The binding of α-helical CAI to HIV-1 CA opens the CA-CTD bundle due to its position in the hydrophobic groove between helices 1, 2, and 4 of the CA-CTD, creating a globular five-helix bundle. The N-terminus of the CAI points inward the bundle, while its C-terminus protrudes out. Thermodynamic characterization of CAI binding to HIV-1 CA protein was examined also by isothermal titration calorimetry [31]. CAI efficiently inhibits the formation of both mature and immature particles in vitro, it destabilizes the CA-CTD-dimer interface and also reduces viral core stability [30].

A major disadvantage of CAI is its impermeability through the cell membranes. Thus, it does not inhibit HIV-1 replication in cell cultures and is therefore not suitable as a potent therapeutic antiretroviral agent.

**NYAD-1 and other CAI derivatives**

To overcome the low permeability of CAI, its structure was subjected to rational design that would stabilize its conformation by intramolecular hydrocarbon stapling, thereby forming the new compound NYAD-1 (Table 1), with increased membrane permeability [32]. The authors retained important CAI residues that interact with the hydrophobic pocket of HIV-1 CA-CTD to preserve the binding site of NYAD-1. By using confocal laser scanning microscopy, NYAD-1 was found to pass through the cell membranes and it was also successfully transported together with Gag to the virus assembly site on the plasma membrane.

NYAD-1 inhibits the in vitro assembly of HIV-1 immature and mature particles in a dose-dependent manner. It prevents the assembly and maturation of the virus, thereby inhibiting the release of virions from infected cells. Although it efficiently inhibits HIV, it is not effective against equine infectious anemia virus (EIAV) or other representatives of lentiviruses [32].

NYAD-13 was created by modifying NYAD-1 (its C-terminal proline was replaced with three lysines) (Table 1) to increase its water solubility [33]. Other CAI-derived stapled peptides are NYAD-36, NYAD-66, and NYAD-67 (Table 1). These peptides have a lower ability to penetrate as NYAD-1, but still retain their ability to disrupt the assembly of HIV-1 CA in vitro. The formed tubular particles are disintegrated in a CAI concentration-dependent manner [34].

**CAC1**

Other peptides that inhibit the assembly of mature HIV-1 are those designed to mimic the CA-CTD helical domains that are involved in intersubunit and intrasubunit interactions. The 20-mer peptide CAC1 (Table 1) was designed to comprise the HIV CA-CTD dimerization helix 9 flanked with additional three amino acids at each end. It binds to CA-CTD and thereby causes dissociation of the CA-CTD dimer of HIV-1, inhibiting CA assembly in vitro [35].

A major disadvantage of the CAC1 peptide is its tendency to aggregate. Therefore, new compounds CAC1-C and CAC1-M (Table 1) have been proposed to improve its solubility and affinity for CA-CTD. CAC1-C originated from CAC1 by substituting Q176, Q179, and E180 for serines and N183 for alanine. Also, CAC1-M was derived from CAC1, by replacement of S178 and Q192 with alanines, A194 for threonine, and adding serine to both ends. Testing showed that despite the fact that these peptides still tend to aggregate they are more soluble and have a higher CA-CTD binding affinity than CAC1. They inhibit the assembly of mature capsids in vitro [35,36].

Not only helix 9, which plays an important role in CA protein dimerization, but also other CA protein helixes, are excellent targets for designing other peptide mimetics of CA protein domains important for the virus assembly. The intermolecular interactions of CA-NTD-CA-CTD in the HIV-1 mature core are mediated primarily by the side chains of helix 8 in the CA-CTD, which packs against the C-terminal end of helix 3 and the N-terminal end of helix 4 in CA-NTD [37]. Therefore, peptides named after these important helices, i.e., H2, H3, H4, and H8, were generated. Of these peptides, only H8 (Table 1) can inhibit the in vitro assembly of HIV-1 CA, but its inhibitory activity is low compared to CAC1 and CAC1-M [36].

Stapled CAC1 peptides were prepared to increase their stability. This approach resulted in two peptides NYAD-201 and NYAD-202 (Table 1). Their binding to the CA-CTD inhibits its dimerization. These two peptides block the virus release in a cell-based assay and the formation of mature-like particles in vitro [38]).

#### 3.2.3. CA-NTD-Binding Non-Peptide Antiretrovirals

**CAP-1**

The public-domain chemical libraries were screened for compounds that bind to the surface of the capsid protein [39]. Forty found compounds with promising effects were tested for binding to CA of HIV-1. This led to the discovery of CAP-1 (N-(3-chloro-4-methyl phenyl)-N’-{2-[({5-[(dimethylamino)-methyl]-2-furyl}-methyl)-sulfanyl]-ethyl}-urea) (Table 1) and CAP-2 (1-(4-(N-methylacetamido)phenyl)-3-(4-methyl-3-nitrophenyl)-urea), which bind to CA-NTD. CAP-2 binds more tightly to HIV-1 CA but, unfortunately, is toxic to cells. NMR analysis revealed that CA undergoes conformational changes after CAP-1 binding. Phe32 is displaced to open the hydrophobic cavity in the CA-NTD, which serves as the binding site.

By measuring HIV-1 CA solution turbidity by spectrophotometric methods, it was found that the rate of in vitro capsid protein assembly is reduced depending on CAP-1 dose and its affinity for the CA. In vivo, already at 100 μM CAP-1 concentration, there was a significant decrease in HIV-1 infectivity of the U1 cells compared to untreated samples. Tang et al. (2003) also showed that CAP-1 does not affect reverse transcriptase or Gag processing and that it does not inhibit virus production. However, the intracellular levels of Gag were lower, depending on the dose of CAP-1 and the authors proposed that this compound could affect intracellular Gag degradation. Moreover, CAP-1 caused morphological changes of cores, as determined by electron microscopy of released virions, suggesting that it also negatively influences the CA-CA interactions and thus the assembly of the mature core. These conclusions suggest that the CAP-1 does not target the early phase event of the virus life cycle, but the late phase event, making it an efficient anti-HIV agent [39].

Although CAP-1 exhibits strong antiviral activity and is not toxic to cells, its affinity for CA-NTD is very low, and therefore it is not applicable for therapeutic use. However, it is a promising candidate for further optimization [40].

**Small Organic Compounds, Acylhydrazones and Their Derivatives**

More than 100 small molecule inhibitors were selected using in vitro CA polymerization assay with an average 50% inhibition concentration (IC50) around 10 µM [41]. Based on their structure several acylhydrazone derivatives were designed and synthesized. Some of these compounds (14f and 14i) (Table 1) showed promising antiviral activities and were able to inhibit HIV-1 assembly in vitro. For these molecules, the hydrophobic cavity of CA-NTD was predicted as a binding site by docking simulation [42,43].

**Benzodiazepines and Benzimidazoles**

Using an in vitro capsid assembly assay [44] to screen a Boehringer Ingelheim corporate compound collection, more than 50 potentially assembly-blocking substances were discovered [45]. Based on criteria such as cytotoxicity assessment of chemical tractability, two large groups of benzodiazepines (BDs) and benzimidazoles (BMs) were selected and further tested.

The basis of BD is 1,5-dihydrobenzo[b][1,4]diazepine-2,4-dione that shows only modest antiretroviral activity itself. Therefore, the analogues of this compound, named BD-1, BD-2, BD-3, and BD-4, were made to improve its inhibitory properties (Table 1). It has been shown that the size and polarity of the substituent on the enamine nitrogen atom, which is important for the inhibition of the capsid assembly, does not influence the antiviral effect of the compound [46].

5-(5-furan-2-yl-pyrazol-1-yl)-1H-benzimidazole was the first BM compound to be discovered, but it did not possess strong antiviral activity. Optimization of the BM structure led to a set of molecules with an increase in its antiviral activity. These modified BMs were named BM-1, BM-2, BM-3, BM-4, and BM-5 (Table 1). As confirmed by NMR spectroscopy and X-ray crystallography, both BD and BM inhibitors bind to CA-NTD of HIV-1. In particular, they bind to a pocket formed by four helices (1, 2, 4, and 7), similarly to a CAP-1 inhibitor [45].

Western blot analysis and electron microscopy revealed that very few HIV-1 virions of different appearance are released from infected cells in the presence of BD, whereas BM does not affect the release of virions, but they are mostly deprived of the cores. Thus, BM appears to inhibit the assembly of mature conical cores [45]. The results show that BD and BM share the same binding site at CA-NTD, but they differ in their mechanisms of action.

The amino acids involved in BM binding are found in the peptidyl-prolyl isomerase CypA binding loop. This CypA binding to the capsid extended loop is important for virus infectivity [47] Goudreau et al. [47] studied whether BD binding also affects the interaction with CypA. Using NMR, they found that BD and CypA bind to CA-NTD simultaneously and do not compete with each other. They also found that binding of inhibitors in the CAP-1 binding site in CA-NTD is possible together with BM binding in the extended helical bundle.

**BMMP**

The yeast membrane-associated two-hybrid assay-based screening of a library containing 20,000 small molecules identified six compounds inhibiting Gag–Gag interactions. Their inhibitory activity was confirmed using the HIV-1 infected T lymphocyte cell line. Compound 172A6 [2-(benzothiazole-2-yl methylthio)-4-methylpyrimidine] (BMMP) (Table 1) targeted HIV-1 CA and prevented the core assembly in vitro. Furthermore, BMMP at high doses affected the post-entry phase of the HIV-1 life cycle by accelerating virus disassembly and uncoating and hence interfered with correct reverse transcription. However, BMMP did not affect the processing of Gag and the release of HIV-1 particles [48]. Contrary to the observed antiviral effect on HIV-1, BMMP showed no effect on SIV and MLV post-entry events [48].

Another study discovered that the binding of biotin to BMMP disrupts its binding to HIV-1 Gag. Quantification of the amount of CA released from the cells showed that the biotin-modified BMMP still had inhibitory activity against the virus, but lower than BMMP alone [49].

**PF74**

A new binding site in CA-NTD of HIV-1 has been discovered in the high-resolution co-crystal structure of HIV-1 CA with newly found antiviral small molecule PF-3450074 (PF74) (Table 1). It was also shown, that the binding of other small molecules to this site could have a strong antiviral effect [50]. PF74 is active against laboratory strains and clinical isolates of HIV-1 in PBMCs, as well as against HIV-2 and SIV. However, it is not effective against other retroviruses such as Bovine immunodeficiency virus (BIV), Feline immunodeficiency virus (FIV), Equine infectious anemia virus (EIAV), N-tropic murine leukemia virus (N-MLV), B-tropic murine leukemia virus (B-MLV), and MoMuLV [43,51].

PF74 binds into an interprotomer binding pocket at the interface of the HIV-1 CA-NTD subunit and the adjacent subunit of CA-CTD, including helices 4, 5, and 7. This binding site is shared also with host cleavage and polyadenylation specificity factor 6 (CPSF6) and nucleoporin 153 (NUP153). The binding of PF74 to HIV-1 CA hexamer is stronger compared to binding of monomeric CA or CA-NTD [52].

Opinions on the mechanisms of action of PF74 are quite diverse and ambiguous. It was published that PF74 affects both the early and late stages of the viral replication cycle. At high concentrations (5–10 μM), it reduces the amount of newly synthesized proviral DNA, causing premature uncoating in infected cells, blocking reverse transcription, and reducing the infectivity of the virus [53]. In vitro experiments have shown that PF74 increases the stability of fusion capsid-nucleocapsid protein (CA–NC) complexes [51], but has destabilizing effects on the already assembled retroviral cores. Thus, it was proposed that PF74 causes premature uncoating of the virus in the cell [43].

**GS-CA1**

GS-CA1 (Table 1), an inhibitor of HIV-1 capsid assembly, was discovered by high-throughput screening of small molecules that bind to HIV-1 CA. It acts on a wide range of HIV-1, HIV-2, and SIV isolates. However, the efficacy against HIV-2 and SIV is reduced compared to HIV-1, possibly due to sequence variability in the GS-CA1 binding site [54]. GS-CA1 has great potential due to its strong antiviral activity against various HIV-1 mutants resistant to non-nucleoside reverse transcriptase inhibitors and the maturation inhibitor. GS-CA1 shows high potency and selectivity in primary human CD4+ T cells and macrophages infected with HIV-1 [54]. Using the crystal structure of the complex of HIV-1 CA with the PF74 inhibitor (mentioned above in PF74) and molecular modeling, it was predicted that GS-CA1 shares the same binding site on the capsid protein as the PF74 inhibitor and host factors CPSF6 and NUP153 [54,55].

GS-CA1 inhibits HIV-1 replication in T cells and peripheral blood mononuclear cells (PBMCs) even at very low concentrations (effective concentration EC50 = 240 pM for T cells and 140 pM for PBMCs) [55].

In vitro treatment with GS-CA1 changes the ordered CA assembly to short and deformed multimers, which differ significantly from the CA tubes formed in vitro at high ionic strength [56,57]. Thus, GS-CA1 disrupts the assembly of capsid protein in vitro. During the early stage of HIV-1 infection, GS-CA1 acts on reverse transcription, interferes with the nuclear import of viral cDNA, decreases the amount of viral DNA in the nucleus of infected cells and retargets it to the cytoplasm of infected cells. During the late stage of HIV-1 infection, GS-CA1 interferes also with the proper release and maturation of viral particles that have variably shaped cores. In vitro testing of GS-CA1 resistance mutations revealed seven important amino acids at the binding site of GS-CA1 L56, N57, M66, Q67, K70, N74, and T107. Viruses containing one or more of these mutations in the capsid protein exhibited reduced susceptibility to GS-CA1 [54].

Due to its high potency, metabolic stability, and low solubility, GS-CA1 is a promising antiretroviral compound. Pharmacokinetic studies in rats have shown that a single subcutaneous injection of GS-CA1 maintains its plasma concentrations well above the effective concentration required for HIV-1 replication inhibition by 95% for more than 10 weeks (paEC95 = 11 nM). This represents a great potential for monthly dosing intervals in humans [58].

The improved version of GS-CA1 designed recently by Gilead Sciences company is compound GS-6207 named Lenapavir (Table 1). It is a first-in-class CA binding inhibitor with long-acting potential [59]. EC50 is 20–160 pM for PBMCs depending on the type of HIV-1 isolate [55]. In a clinical study in healthy people, single subcutaneous doses of GS-6207 up to 450 mg were well tolerated and maintained systemic exposure for over 24 weeks [60]. This drug is currently being tested in HIV-positive people [61].

The data published by Link et al. [62] suggest that GS-6207 binds to conserved residues in the interface between CA monomers similarly to PF74 and blocks multiple steps in the virus replication cycle; especially the nuclear import of HIV-1 core by competitive binding of CA with nuclear import cofactors of the infected cell; especially NUP153 and CPSF6. This has been recently supported by the data of Zila et al. [63] that demonstrate that whole HIV-1 core enters the nucleus and this process depends on the binding of CA to these factors. GS-6207 thus prevents the uncoating of HIV-1 core. Clinical studies have shown that GS-6207 can dramatically decrease the virus load after a single subcutaneous dose. Its remarkable stability predestines this inhibitor for a long-term application [62].

**BI-1 and BI-2**

A phenotypic cell-based viral replication assay using a screening of a corporate (private) library of ~60,000 compounds led to the discovery of a new family of 4,5-dihydro-1H-pyrrolo[3,4-c]pyrazole-6-one (pyrrolopyrazolone) compounds named BI-1 and BI-2 [64] (Table 1). These compounds acted during early post-entry events and not during the late phase of the HIV-1 replication cycle. The qPCR analysis of accumulated late reverse transcription products and 2-LTR circles in infected cells treated by BI-1 showed that BI-1 does not affect reverse transcriptase activity (the same applies for BI-2) and late reverse transcription production. However, it reduces the concentration of 2-LTR circles suggesting that both BI’s inhibit nuclear import of the pre-integration complex [64].

In vitro experiments have shown that BI-1 assists in the assembly of CA–NC HIV-1 by binding to HIV-1 CA-NTD and thereby stimulates the assembly of CA hexagonal lattice. This binding also inhibited the disassembly of CA–NC complexes [64]. In another study, it was shown that BI-2 destabilizes the HIV-1 core during infection [65]. The fact that both BIs facilitate the in vitro assembly of HIV-1 CA–NC complexes and simultaneously destabilize the core of the virus can be explained in two ways. First, CA–NC complexes consist predominantly of hexamers in vitro, while the retroviral core consists of both hexamers and pentamers [66,67]. BIs can affect the flexibility of the capsid and thereby primarily promote the formation of CA protein hexamers. Second, the presence of cellular factors is required to destabilize CA–NC complexes in vitro [65]. Another approach to confirming these results was achieved by testing the ability of BI-2 to destabilize the CA–NC complexes of HIV-1 in vitro, in the presence of cell extracts. The results showed that the presence of cell extracts did not affect the BI-2-mediated destabilization of the CA–NC complexes [51].

Isothermal titration calorimetry and subsequent analysis of NMR chemical shifts of HIV-1 CA protein induced by the addition of BI-2 were used to map the binding site of BI inhibitors. It was found that BI-2 inhibitor binds to the same binding pocket formed by helices 4, 5, and 7 in CA-NTD, as previously described as the PF74 inhibitor. Experiments revealed that in the case of HIV-1, the same inhibitory effect was achieved with 50 μM BI-2 and 5 μM PF74 [65]; however, with a different mechanism of action. BI-2 acts only during the early phases of the replication cycle, while PF74 acts also in its late phase. In contrast to PF74, BI-2 does not affect reverse transcription in HIV-1 infected cells. Moreover, BI-2 stabilizes CA cores, while PF74 causes their dissociation [64].

The BI and host factors that affect HIV-1 activity share the same binding site. Thus, BI competes with these factors for binding to HIV-1 CA as shown for CPSF6, which is important for HIV-1 infection because its binding to CA enables active nuclear import of the core [68]. The fact that the binding of BI to HIV-1 CA protein inhibits its interactions with host proteins was further confirmed by Lamorte et al. [64]. Both BI-2 and PF74 prevent the binding of CPSF6 not only to CA–NC complexes of HIV-1 but also to SIV. A 50 μM concentration of BI-2 completely inhibits the binding of CPSF6 to the in vitro assembled HIV-1 CA–NC complexes [65].

BI-2 has been shown to block both HIV-1 and SIV infection but did not affect HIV-2, BIV, FIV, EIAV, N-MLV, B-MLV, and Mo-MLV [65]. Since BI-2 is not toxic to the cells, it has a good potential to serve as an efficient antiviral agent.

**Phenylalanine Derivatives 11l and Q-c4**

Design and synthesis of 37 novel benzenesulfonamide-containing phenylalanine derivatives, obtained by structural modifications of PF74, led to the discovery of a new, very potent HIV-1 and HIV-2 inhibitor named 11l [69] (Table 1). This compound binds to HIV capsid at the same site as PF74 but shows approximately six-fold higher activity than PF74 against HIV-1. EC50 values of 11l and PF74 were determined as 90 and 520 nM, respectively. The authors further proposed that the mechanisms of action of 11l and PF74 differ. 11l inhibits the virus at both the early and late stages of the virus life cycle, in the early stage probably by accelerating virus particle uncoating, which is incompatible with reverse transcription, and in the late stage by altering the assembly of the immature particles [69]. Because 11l is not acutely toxic even at very high doses, it may be a very promising antiretroviral drug for further testing, optimization, and clinical use.

Another series of phenylalanine derivatives covalently cross-linked into dimers by using 2-piperazineone or 2,5-piperazinedione was shown to possess higher activities than phenylalanine-derived monomers. Although the most potent inhibitor in this series, Q-c4 (Table 1), showed slightly lower binding affinity to HIV-1 CA than the control molecule PF74, its antiviral activity was comparable with PF74 [70]. It exhibited an EC50 value of 0.57 μM in the infected MT-4 human T-cell line. Q-c4 binds to the same capsid interprotomer pocket as PF74 and thereby prevents binding of HIV-1 CA with the cellular factors CPSF6 and NUP153. It affects the HIV-1 life cycle both at early and late stages, dominating in the late stage of the viral life cycle [70].

**CK026 and I-XW-053**

Iterative in silico-in vitro hybrid structure-based virtual screening [71] was used to identify new small molecules that can interact with HIV-1 CA-NTD and inhibit the NTD-NTD interaction interface, thus affecting the early steps of the HIV-1 replication event. This approach led to the discovery of a small molecule 4,4′-[dibenzo[b,d]furan-2,8-diylbis(5-phenyl-1H-imidazole-4,2-diyl)]dibenzoic acid (CK026) (Table 1) with anti-HIV-1 activity. Three analogues of this molecule were further synthesized to improve its properties. Of these three, only the compound named I-XW-053 (4-(4,5-diphenyl-1H-imidazol-2-yl)benzoic acid) (Table 1) still retained its potent anti-HIV-1 activity, although showing no activity against SIV or other retrovirus unrelated viruses. The results obtained from docking studies, confirmed by isothermal titration calorimetry and surface plasmon resonance showed that I-XW-053 binds to CA-NTD in a 1:2 ratio. Since the authors demonstrated that I-XW-053 disrupts HIV-1 CA assembly in vitro and also inhibits reverse transcription in multiple cell types, they concluded that this compound very likely affects the virus uncoating process [72].

**Compound 696**

A screening of an in-house library of 400 compounds identified a feline immunodeficiency virus (FIV) assembly inhibitor, named **696** (Table 1) [73]. The authors also identified the binding pocket within FIV CA where **696** binds. Despite the rather low affinity of this inhibitor that is around 100 µM, the authors propose to use it as a lead compound for further optimization of FIV inhibitors, as the HIV-1 assembly inhibitors are usually inactive against FIV.

#### 3.2.4. CA-CTD-Binding Non-Peptide Antiretrovirals

**Compounds 6 and 55**

The hydrophobic cavity in HIV-1 CA-CTD has been previously described as a possible target for peptide-based inhibitors. By screening 100,000 substances from the ZINC database [74], 200 substances were selected, and 50 substances interacting with the hydrophobic cavity were identified by further in silico testing using 3D stereoscopic glasses. Authors found that compounds named Compound **6** and **50** (Table 1) prevent the formation of mature-like particles in vitro in a dose-dependent manner. Treatment of virus-producing cells with compounds **6** and **55** (analogue of 50) (Table 1) reduced the production of virions, which were also much less infectious, suggesting that these compounds interfere with viral uncoating or assembly/maturation, thus causing the production of damaged virions [75].

**Ebselen**

An organoselenium compound ebselen (2-phenyl-1,2-benzisoselenazol-3(2H)-one) (Table 1) was selected from the library of 1280 pharmacologically active drugs in vivo by high-throughput screening method based on time-resolved fluorescence resonance energy transfer (HTS-TR-FRET), assessing HIV-1 CA-CTD dimerization [76]. Liquid chromatography–electrospray ionization mass spectrometry confirmed the binding of ebselen to HIV-1 CA-CTD. It was found that cysteine residues Cys198 and Cys218 are important for the binding of ebselen to the CA-CTD of HIV-1 through the formation of a covalent bond with a selenium atom resulting in a selenylsulfide linkage [77].

Ebselen thus influences the early phase of the retroviral life cycle by disrupting capsid uncoating. It functions in a dose-dependent manner by affecting reverse transcription, uncoating process, and stabilization of mature capsid.

Exceptionally, among the abovementioned inhibitors, ebselen reduces not only the infectivity of HIV-1 but also of other retroviruses such as Moloney murine leukemia virus (MoMuLV) and Simian immunodeficiency virus (SIV). However, it does not show an inhibitory effect on hepatitis and influenza virus [76].

**Other Inhibitors**

The fluorescent-based method [78], which is used to detect and visualize capsid protein interactions, e.g., CA protein dimerization, has also been shown to be useful for testing possible inhibitors of assembly of the capsid protein in vitro. Screening of the library containing 1280 pharmacologically active substances revealed four substances significantly inhibiting the dimerization of CA-CTD: taurocholic acid (TA) (Table 1), aminoguanidine hemisulfate (AGH), (±)-2-amino-5-phosphonopentanoic acid (A5PA), and acetylsalicylic acid (ASA). Further tests used to clarify whether these agents inhibit the assembly of HIV-1 in vitro showed that only TA successfully inhibits the CA-CTD dimerization in a dose-dependent manner. Three commercially available derivatives of TA: glycocholic acid (GCA), taurodeoxycholate (TDC), and glycodeoxycholate (GDC) (Table 1) were also tested for their inhibitory effect. GDC was proven to be less toxic to cells than the previously described CAP-1 inhibitor (decreased viability by 19% at 200 μM GDC in contrast to more than 50% at 200 μM CAP-1). The mechanism of action of GDCs is in disruption of Gag–Gag interactions by blocking the CA protein mutual interactions [78].

Homogeneous time-resolved fluorescence (HTRF) technology has been invented as a new method for detecting small substances that target the same CA binding site as the abovementioned CAI. Screening of the library with 464 protein kinase inhibitors led to the identification of TX-1918 (Table 1), which inhibited the processing of Gag polyprotein in a dose-dependent manner. It also inhibited the HIV-1 CA assembly in vitro as observed by changes in absorbance [79].

#### 3.2.5. CA-SP1 Binding Antiretrovirals

**Bevirimat**

Betulinic acid (Table 1), a triterpenoid isolated from leaves of *Syzygium claviflorum*, has been shown to inhibit HIV-1 replication [80]. Rationally-directed modification to improve its antiviral activity resulted in 3-O-(3′, 3′-dimethylsuccinyl)betulinic acid (Bevirimat, PA-457) (Table 1), which inhibited both HIV-1 and also its protease inhibitor-resistant forms. Bevirimat binds to and stabilizes the six-helix bundle of HIV-1 fusion capsid protein-spacer peptide (CA-SP1) [81,82], thus inhibiting its proteolytic processing. Surprisingly, bevirimat is active only on assembled Gag and not on free monomeric Gag, which must be considered when testing its effects in vitro [82].

Bevirimat-mediated inhibition of HIV-1 maturation results in aberrant morphology of viral cores, which are spherical, acentric and the virus particles released from bevirimat-treated cells also often contain an electron-dense layer in the viral membrane [83].

Bevirimat was the first drug tested to inhibit HIV-1 maturation. Animal studies have shown that bevirimat is rapidly absorbed after intravenous or oral administration and quickly spreads to peripheral tissues [84]. Phase I and II clinical trials have shown that the drug has been well-tolerated, has a long half-life and potency, has no side effects, and reduces the viral load [85]. However, research has shown that some patients had resistant viruses with naturally reduced sensitivity to this drug and did not respond to treatment. Genotypic analysis of HIV-1 mutants isolated from these patients revealed that low response to treatment is associated with a polymorphism at positions Q369, V370, and T371 in HIV-1 SP1 [86,87,88].

#### 3.2.6. Inhibitors Binding to Assembly Intermediates

Based on a cell-free HIV-1 assembly screening, Reed et al. [89] have recently identified a small tetrahydroisoquinolone derived inhibitor of HIV-1 assembly PAV-117 (Table 1). Its analogue PAV-206 (Table 1) showed more potent activity. They also reported that this small molecule binds to a multiprotein complex that contains Gag assembly intermediates, ATP-binding cassette protein E1 (ABCE1), and DEAD box RNA helicase 6 [89].

#### 3.2.7. Antibodies

The HIV-1 CA protein represents also a promising target for antibodies. Monoclonal antibodies are stable and provide the attractive potential for in vivo applications. Previous experiments have shown that monoclonal antibodies are capable of inhibiting capsid protein polymerization in vitro [90]. To overcome their poor penetration through the cell membranes, antibodies can be conjugated to various cell-penetrating peptides [91,92]. This modification was tested by evaluating the ability of antibodies to enter T cells and inhibit HIV-1 replication. The cell uptake was time-dependent and reached a plateau at 18 h after the exposure.

The conjugation of antibodies with a short membrane transport sequence (KGEGAAVLLPVLLAAPG) improved the antibody internalization [93]. Later studies showed that κFGF-MTSs and HIV-R9-Tat protein were the most permeable and κFGF-MTS attached to the antibody against HIV-1 CA targeted early stages of viral infections and efficiently inhibited the virus replication both in T cell lines and primary PBMCs [91].

**Table 1:** List of compounds binding to retroviral capsid protein.

Compound	Structure	Inhibition Efficiency	Ref.
CAI	ITFEDLLDYYGP	N.D.	
NYAD-1	ITFXDLLXYYGP, X represents [(S)-2-(2′-pentenyl) alanine]	EC5O 4.29−21.6 µM	[32]
NYAD-13	ITFXDLLXYYGKKK, X represents [(S)-2-(2′-pentenyl) alanine]	N.D.	
NYAD-36	ISF-R8-ELLDYY-S5-ESGS, S5 represents [(S)-2-(4′-pentenyl)alanine] and R8 [(R)-2-(7′-octenyl)alanine]	EC5O 1.5 ± 0.7 µM	[34]
NYAD-66	ISF-R8-ELLDYY-S5-ED, S5 represents [(S)-2-(4′-pentenyl)alanine] and R8 [(R)-2-(7′-octenyl)alanine]	EC5O 3.94 ± 0.32 µM	[34]
NYAD-67	ISF-R8-EWLQAY-S5-EDE, S5 represents [(S)-2-(4′-pentenyl)alanine] and R8 [(R)-2-(7′-octenyl)alanine]	EC5O 3.88 ± 0.3 µM	[34]
CAC1	EQASQEVKNWMTETLLVQNA	N.D.	
CAC1-C	ESASSSVKAWMTETLLVQNA	N.D.	
CAC1-M	SESAASSVKAWMTETLLVANTSS	N.D.	
H8	KEPFRDYVDRFYKTLRAEQ,	N.D	
NYAD-201	AQEVKXWMTXTLLVA, X represents [(S)-2-(2′-pentenyl)alanine]	N.D.	
NYAD-202	AQAVKXWMTWTLLVA, X represents [(S)-2-(2′-pentenyl)alanine]	N.D.	
CAP-1	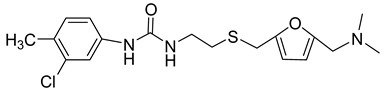	EC95 ≈100 µM	[39]
14f	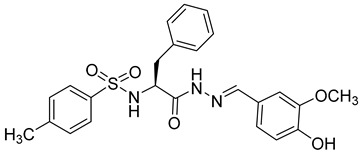	EC5O 0.21 µM	[42]
14i	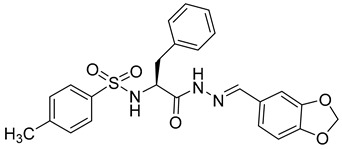	EC5O 0.17 µM	[42]
BD-1	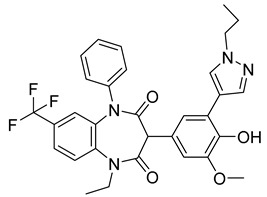	EC50 70 ± 30 nM	[45]
BD-2	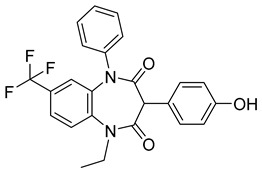	EC50 1.1 µM	[45]
BD-3	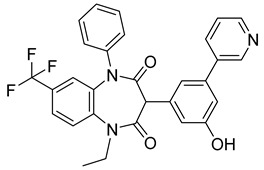	EC50 0.43 µM	[45]
BD-4	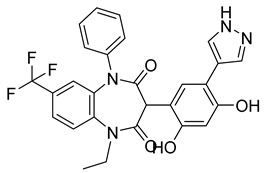	EC50 0.13 µM	[45]
BM-1	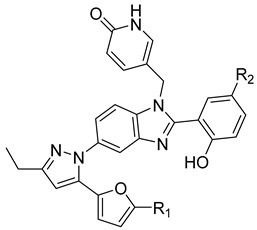	EC50 62 ± 23 µM	[45]
BM-2	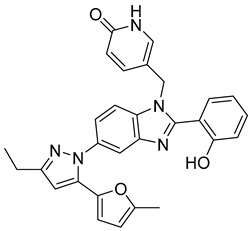	EC50 0.26 µM	[45]
BM-3	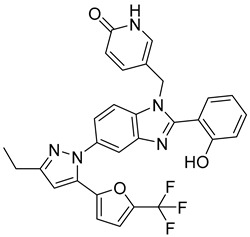	EC50 0.11 µM	[45]
BM-4	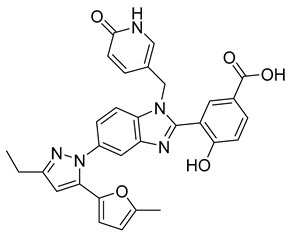	EC50 46 µM	[45]
BM-5	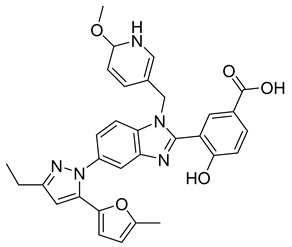	EC50 2.4 µM	[45]
BMMP	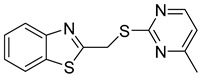	N.D.	
PF74	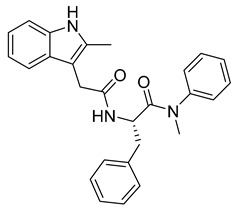	EC50 8−640 nM	[50]
GS-CA1	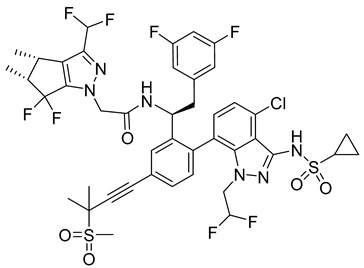	EC50 140−240 pM	[55]
GS-6207 (Lenapavir)	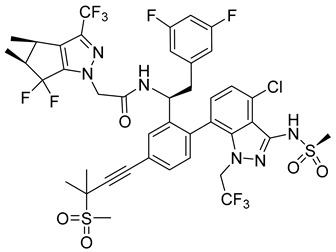	EC50 50−100 pM	[55]
Bl-1	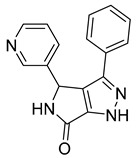	EC50 7.5 ± 2.1 µM	[64]
Bl-2	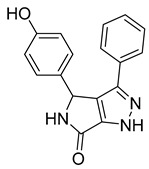	EC50 1.4 ± 0.66 µM	[64]
11l	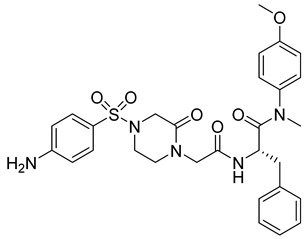	EC50 90 nM	[69]
Q-c4	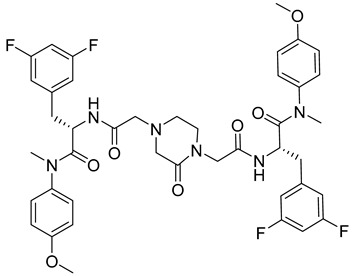	EC50 0.57 µM	[70]
CK026	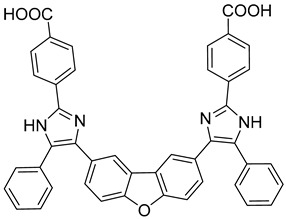	N.D.	
I-XW-053	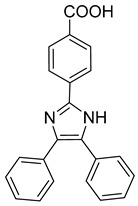	EC50 100 µM	[72]
696	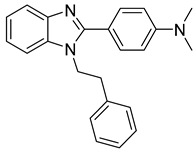	N.D.	
Compound **6**	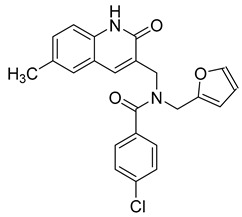	EC50 1.6−6.17 μM	[75]
Compound **50**	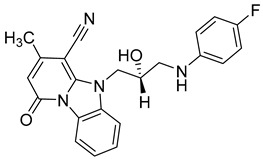	EC50 1.12−10.95 μM	[75]
Ebselen	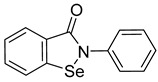	EC50 1.99 ± 0.57 μM	[76].
TA	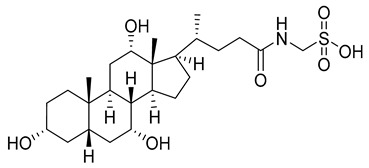	N.D.	[78]
GDC	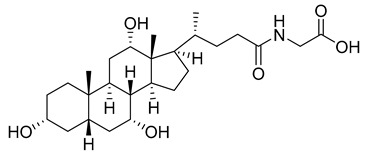	EC50 >200 µM	[78]
TX-1918	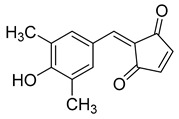	EC50 15.16 µM	[79]
Betulinic acid	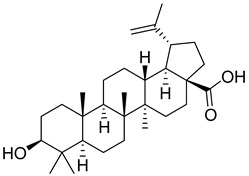	EC50 1.4 µM	[94]
Bevirimat	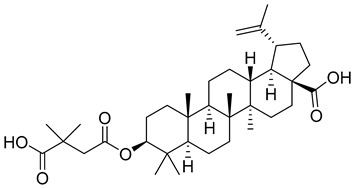	EC50 3.5 nM	[94]
PAV-117	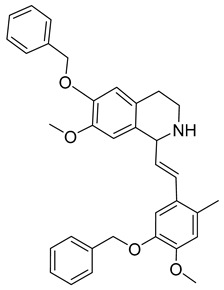	EC50 48 nM	[89]
PAV-206	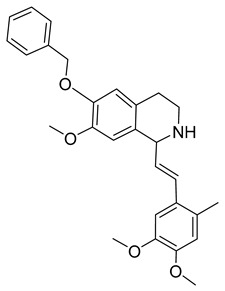	EC50 36 nM	[89]

## 4. Hepatitis B Virus Assembly Inhibitors

Despite available well-functioning vaccines and inhibitors targeting various steps of the virus life cycle (recently reviewed in [95]), hepatitis B-virus (HBV), an enveloped DNA virus, remains a major health problem [96]. HBV replication depends on a step catalyzed by error-prone reverse transcriptase, and has therefore been included among RNA viruses with low fidelity polymerases in this review. HBV is a causative agent of chronic liver diseases, which may ultimately lead to cirrhosis or hepatocellular carcinoma. The WHO fact sheet says that: “in 2016, 27 million people (10.5% of all people estimated to be living with hepatitis B) were aware of their infection”. The life cycle of HBV has recently been reviewed [97]. HBV is an enveloped DNA virus that assembles from core protein molecules present in homodimeric subunits into icosahedral T = 4 HBV capsid consisting of 180–240 copies of the core proteins (HBc or named Cp) [98]. The HBc protein is the capsid-forming protein that primarily dimerizes with some structural plasticity [99] manifested by different inter-dimer interfaces of the HBc homodimers named AB, CD, or EF dimers [100]. Most of the published compounds bind to such hydrophobic interfaces and interfere with the assembly of virus particles [101]. Besides these so-called Dane infectious spherical particles of 42 nm in diameter [102], also smaller spherical and filamentous structures with a diameter of 22 nm may be found in the sera of infected individuals. In contrast to the Dane particles, the smaller forms do not contain viral RNA and are thus non-infectious. The multiple roles of the core protein including possible ways of intervention that block the HBV core protein dimerization, capsid assembly, or disassembly have been nicely reviewed [103,104,105].

In the last two decades, numerous inhibitors targeting the HBV core protein have been designed. Three structural groups of core protein allosteric modulators are represented primarily by three groups of compounds phenylpropenamides, heteroaryldihydropyrimidines, and sulfamoyl benzamides [106,107,108,109,110,111] (Table 2). According to the mechanism of action, allosteric modulators have been divided into two groups. In type I, some compounds cause misassembly of capsids, resulting in the formation of either aberrant non-infectious particles or protein aggregates. In contrast, type II modulators result in the assembly of virus-like particles with normal morphology but without genomic RNA [112]. Representatives of both groups are currently in clinical trials (for review see [103]).

Importantly, some of these inhibitors block the propagation of HBV mutants resistant to nucleoside analogues [113]. This is a desirable benefit of inhibitors with another mode of action than the frequently used efficient inhibitors of virus genome replication represented mostly by modified nucleosides. Interestingly, acceleration of the assembly by phenylpropenamides may have a similar phenotypic effect in terms of blocking the virus propagation as the heteroaryldihydropyrimidines that induce the assembly of aberrant non-capsid aggregates [114].

There exists a series of non-nucleoside substituted propenamide derivatives that interfere with the packaging of the viral pregenomic RNA and assembly of immature HBV core particles [114,115]. HAP_R01 (Table 2) which is the member of heteroaryldihydropyrimidine core protein allosteric modulators directly triggers misassembly of HBV [114]. Interestingly, this propenamide derivative binds to the same pocket as the SBA_R01 inhibitor (also titled NVR 3-778) (Table 2) that is representative of the sulfamoylbenzamide group of inhibitors [116]. The structural analysis identified that besides the main binding pocket, there is a unique hydrophobic subpocket in the dimer–dimer interface of HBV core protein hexamer, where fits the thiazole group of HAP_R01, but it is unperturbed by SBA_R01 [116]. The authors concluded that such different mechanisms of inhibitors acting at the same site may help to design suitable molecules to counter the potential resistance of HBV. SBA_R01 is currently in clinical development [117,118]. Moreover, the efficacy of this inhibitor was enhanced by its co-administration with pegylated interferon α [117].

Another orally administered inhibitor is an allosteric modulator of HBV core protein, titled RO7049389 (RG7907) (Table 2), which causes defective capsid assembly [119]. This inhibitor, developed by Roche, showed favorable safety, tolerability, and pharmacokinetics profile and has been classified as suitable for further clinical development [120]. This compound was also further successfully modified by installing functional moieties to increase the inhibitory activity of SBA_R01 as proved for sulfamoylbenzamide-based compound KR-26556 [121] (Table 2). Especially modifications of positions 4 and 6 with fluorine and methoxy group, respectively, resulted in significant improvement of the inhibitor potency.

The KR-26556 was subjected to further optimization. It was found that the 3,4-difluoro compound is more efficient than the monofluorinated one and changes the conformations of the AB dimer of the HBc protein and the degree of its α-helix flexibility [122]. The transmission electron-microscopic analysis showed that this change induces the formation of aberrant tubular particles. In contrast, the use of some sulfamoylbenzamides, developed in the Schinazi group, led to the formation of aberrant spherical particles that readily aggregated [123]. Another molecule, GLP-26 (Table 2) a glyoxamide derivative, showed high inhibitory potential against HBV capsid assembly [124]. This compound significantly reduced viral replication in an HBV nude mouse model bearing HBV transfected AD38 xenografts [125]. Very promising is the combination therapy using GLP-26 and entecavir. GLP-26 is currently being offered as an HBV capsid assembly modulator by MedChemExpress.

The structure hopping and structure-activity relationship (SAR) studies combined with the high throughput screening (HTS) resulted in the identification of a 2-aminothiazole based inhibitor that exhibited anti-HBV replication activity in vitro [126]. Structure modification and SAR led to improved inhibitory efficacy of the sulfamoylbenzamide class compound SBA_R01 (NVR 3-778) (Table 2), which is in phase II clinical trial for the treatment of HBV infection [127]. Similar approaches were used to optimize other compounds; e.g., tetrahydropyrrolopyrimidines based on Bay41_4109 and GLS4 [128], N-phenyl-3-sulfamoyl-benzamide derivative inhibiting HBV capsid assembly in vitro [129] (Table 2), or to explain the enhanced binding of heteroaryldihydropyrimidine inhibitor to the Y132A mutant compared to the wild type of the core protein [130]. 4-oxotetrahydropyrimidine-derived phenyl urea, compound 27 (58031) (Table 2), and some analogues were shown to inhibit HBV by mistargeting the HBc protein to assemble empty capsids devoid of viral pregenomic RNA in human hepatocytes [131]. Jia et al. [132] used molecular modeling based on scaffold hopping and bioisosterism for rational modification of previously published lead compounds isothiafludine (NZ-4) [133] and AT-130 [134] (Table 2). The in silico screening revealed several pyrimidotriazine derivatives, which inhibited HBV assembly both in HepG2.2.15.7 cells and in the in vitro system using TnT^®^ Coupled Reticulocyte Lysate System (Promega, Fitchburg, WI) [135].

Using SAR, a new group of HBV assembly modulators based on pyrazolo piperidine scaffold was identified by Kuduk et al. [136]. They found that the attachment of a 6-Me group (S-configuration) increased both the efficiency and metabolic stability of the compound.

Recent clinical studies indicate that core protein (Cp) allosteric modulators may inhibit Cp dimer–dimer interactions not only by interfering with the nucleocapsid assembly and viral DNA replication but also by inducing the disassembly of double-stranded DNA-containing nucleocapsids to prevent the synthesis of cccDNA. Another docking-based virtual screening led to the discovery of 2-aryl-4-quinolyl amide derivate (II-2-9) (Table 2), which blocks the HBV capsid interactions [137].

Recently, by screening an in-house compound library, a new molecular motif; N-(4-nitrophenyl)-1-phenylethanone hydrazone (Table 2) was identified as the inhibitor targeting HBV replication by targeting its capsid assembly [138]. This compound accelerates the assembly and induces the formation of empty viral particles lacking genomic RNA.

**Table 2:** List of hepatitis B virus assembly inhibitors.

Compound	Structure	Inhibition Efficiency	Ref.
Bay 41-4109	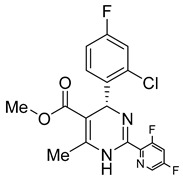	EC50 0.12 ± 0.026 µM	[139]
GLS4	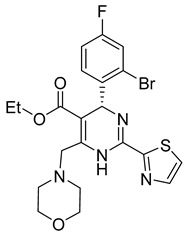	EC50 15 ± 5.3 nM	[139]
HAP_R01	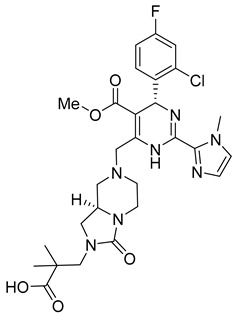	EC50 1.12 µM	[114]
AT-130	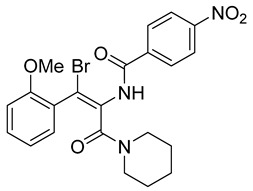	EC50 0.13 µM	[140]
SBA_R01 (or NVR 3-778)	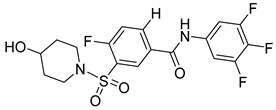	EC50 0.36 ± 0.015 µM	[121]
KR-26556	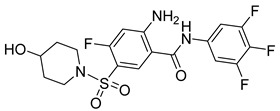	EC50 40 nM	[121]
RO7049389 (RG7907)	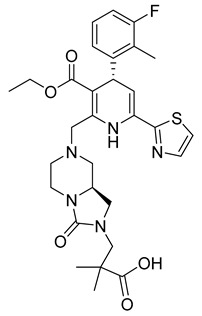	EC50 6.1 ± 9 nM	[119]
GLP-26	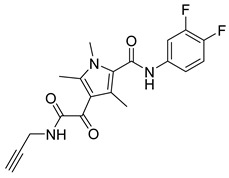	EC50 3 nM	[141]
Compound **27** (58031)	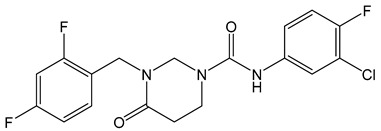	EC50 0.52 µM	[141]
NZ-4	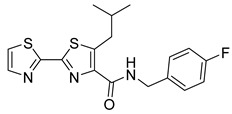	EC50 1.33 µM	[141]
II-2-9	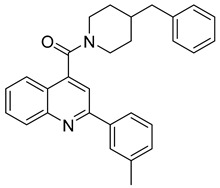	EC50 1.8 µM	[141]
N-(4-nitrophenyl)-1-phenylethanone hydrazone	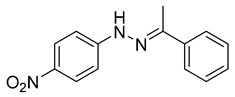	N.D.	

## 5. Flavivirus Assembly Inhibitors

### 5.1. Hepatitis C Virus Assembly Inhibitors

Hepatitis C virus (HCV), a member of flaviviruses, is an enveloped RNA virus encoding one polyprotein precursor of ten proteins of mature virus that are released by the action of both cellular and viral proteases [142]. The assembly of HCV remains only partially understood. It occurs at the endoplasmic reticulum and yields a heterogeneous population of pleomorphic mostly non-infectious particles [143]. It is triggered by the interaction of NS5A protein with mature 21 kDa core protein, which consists of three domains. The N-terminal, basic domain binds viral RNA, the second domain is hydrophobic and less basic, which is optimal for binding to lipid droplets and the last domain contains the signal sequence for the ER membrane translocation. The mechanisms of specific incorporation of viral genomic RNA and regulation of recruitment of subpopulation of lipoproteins and apolipoproteins and heterodimers of HCV E1 and E2 envelope glycoproteins is still poorly explained.

The HCV infection may cause similar chronic liver diseases as mentioned above for HBV, although some HCV infections are either asymptomatic or manifested as acute hepatitis which does not represent a life-threatening disease. However, approximately two-thirds of infected individuals develop chronic HCV infection with a 20% risk of developing cirrhosis within 20 years of infection, which remains a global public health problem [144].

The combination of ribavirin with pegylated interferon α was the preferred anti-HCV therapy, however, its high cost, rather low efficacy together with serious side effects in some patients limit its use [145,146]. Current anti HCV therapy is based on nucleo(s)tides inhibitors (e.g., sofosbuvir) targeting mostly NS5B protein which is an RNA-dependent RNA polymerase or NS5A protein, which is an essential component of NS5B and modulates viral replication and other processes [147,148,149]. A major concern connected with the use of these direct-acting antivirals is rapid emergence of drug-resistant variants of HCV due to a poor fidelity of the RNA dependent RNA polymerase [146]. Other efforts are focused on NS3 serine protease and its associated helicase activity [150]. It is known that HCV employs lipid remodeling to increase its replication efficiency [151]. Avasimibe (Table 3), a lipid-lowering drug that inhibits acyl coenzyme A:cholesterol acyltransferase specifically inhibited assembly of HCV by downregulating microsomal triglyceride transfer protein responsible for the formation of very-low-density lipoproteins (VLDL) and chylomicrons which are important for the HCV assembly [152,153]. Another HCV assembly inhibitor acting through modulation of lipid metabolism is glycogen synthase kinase 3β inhibitor [154]. The human heat shock cognate protein 70 (Hsc70) appears to be also a host cell protein whose inhibition specifically blocks the HCV assembly [155,156,157].

Extensive screening has identified several promising inhibitors that bind to the core protein and potentially inhibit the assembly of HCV particles. Successful drug, daclatasvir (methyl N-[(2S)-1-[(2S)-2-[5-[4-[4-[2-[(2S)-1-[(2S)-2-(methoxycarbonylamino)-3-methylbutanoyl]pyrrolidin-2-yl]-1H-imidazol-5-yl]phenyl]phenyl]-1H-imidazol-2-yl]pyrrolidin-1-yl]-3-methyl-1-oxobutan-2-yl]carbamate) (Table 3) was approved in 2014 by the European Medicine Agency and in 2016 by the FDA, under the brand name Daklinza. It is usually co-administered with sofosbuvir [158]. It was proposed that daclatasvir blocks the transport of viral RNAs to the assembly sites and thus inhibits viral particle assembly [159].

High throughput screening of about 350,000 compounds in a human hepatoma-derived cell line, Huh7.5.1, a suitable infection system for hepatitis C virus, yielded 158 molecules with anti-HCV activity [160]. Later focus on their mechanism of action resulted in the identification of 6-(4-chloro-2-methylphenoxy)pyridin-3-amine (Table 3), which inhibited the viral morphogenesis [161]. Indirect inhibition of assembly was achieved by inhibition of AAK1 and GAK, serine/threonine kinases stimulating binding of clathrin adaptor protein complex 2 to cargo, and are essential for HCV assembly [162]. Fluoxazolevir, an aryloxazole-based compound (Table 3) was shown to inhibit entry of hepatitis C virus by binding to HCV envelope protein 1 and preventing fusion [163].

Repurposing already approved drugs seems to be a promising approach also in the treatment of HCV infection. The FDA-approved antihistamine drug chlorcyclizine hydrochloride (Table 3) was found to inhibit some of the late steps of the HCV life cycle, but not likely the assembly [164]. Its efficacy was proved in chimeric mice engrafted with primary human hepatocytes infected with HCV [165].

The same laboratory has recently published 4-aminopiperidine-based compounds that inhibit HCV assembly [166]. Moreover, the effect was synergistic with some FDA-approved drugs as broad spectrum antivirals; cyclosporin A and ribavirin or telaprevir and daclatasvir [166].

Kota et al. [167] developed the assay for monitoring the dimerization of the HCV core capsid protein, a process that is essential for the formation of virus particles. By the use of capsid protein-derived peptides, they identified two 18-residue peptides that bound to core protein and thus efficiently inhibited its dimerization. These two peptides were also tested on HCV-infected cells but did not show activity to block virus replication, they affected only the virus release. Later, these authors used HTS-TR-FRET for screening of two large libraries, LOPAC, consisting of 1280 molecules, and Boston University library with 2240 compounds to inhibit HCV capsid protein dimerization. Of the 28 positive hits selected, a molecule named SL201 (Table 3) was also tested on the Huh-7.5 cell line infected with HCV 2a J6/JFH-1 strain and found to be effective at micromolar concentration [168]. Thus this molecule was used for its further elaboration together with another three hits from another library screening (molecules were labelled 1–4 [169]). By further modification and dimerization of the most active molecule 2, two indoline alkaloid–type promising inhibitors with IC50 in the nanomolar range (Table 3), were successfully synthesized. In another study, molecules 1 and 2 were biotinylated and tested for their efficient entry into HCV-infected cells where they associated with HCV core [170].

### 5.2. Dengue Virus Inhibitors

Dengue viruses (DENV) belong to the *Flaviviridae* family. With almost 400 million infections worldwide they represent one of the main global health burdens. The precise global incidence of dengue fever is uncertain and regional outbreaks occasionally occur. In 2013, it was published that about one third of the human population is at risk of dengue virus infection, with approximately 390 million cases reported per year [171]. This situation is becoming even more serious due to the recently reported large number of dengue-positive animal species and thus a risk of enzootic cycle. The WHO reported 5.2 million cases in 2019, of which about 3.1 million cases were in the American region [172].

Since DENV exist in four distinct serotypes, the development of an efficient vaccine has been very challenging and quite recently, the first vaccine, CYD-TDV (sold under the brand name Dengvaxia^®^) was approved by the FDA. Although several effective molecules targeting various steps of DENV replication machinery have been identified (reviewed in [173]), there are still no available potent antiviral therapeutics on the market. The need to find new DENV antivirals is therefore very important. A potent antiviral molecule named ST-148 that targets the capsid protein of DENV was discovered by the HTS of approximately 200,000 chemically diverse compounds by Byrd et al. [174] (Table 3). It inhibits all four serotypes of DENV by binding to its capsid core C protein. ST-148 has a potent in vitro and in vivo activity in low micromolar concentrations (ranging from 0.016 to 2.832 μM) and acts in various cell types. Importantly, it is not cytotoxic. Its exact mode of action on DENV capsid protein was further studied by a combination of several biochemical, virological and imaging-based techniques by Scaturro et al. [175]. The authors found that binding of ST-148 to capsid protein enhances capsid–capsid interactions, stabilizing virus nucleocapsid and thus affecting both the assembly and disassembly of this virus.

A new compound, 3-amino-6-phenyl-N-(4-phenyl-1,3-thiazol-2-yl)-5,6,7,8-tetrahydrothieno[2,3-b]quinoline-2-carbox-amide (named VGTI-A3) (Table 3) was discovered by HTS of more than 5600 molecules for their effect on DENV-infected cells [176]. Despite its high antiviral activity, it exhibited low solubility and thus a series of its analogues were synthesized. By testing their activities, the compound named VGTI-A3-03 was selected [177] (Table 3). It exhibited a strong antiviral effect not only against all DENV serotypes but also against West Nile virus. Due to its similar structure to the previously found molecule ST-148 (see the text above), it was predicted to have the same or similar mode of action, by binding the capsid protein of DENV. Unfortunately, VGTI-A3-A3 resistant mutants appeared after only a few passages of the DENV infected cells treated with this inhibitor. The resistance-causing mutation was found to be located in the region of DENV capsid protein [177].

**Table 3:** List of flavivirus assembly inhibitors.

Compound	Structure	Inhibition Efficiency	Ref.
**Hepatitis C virus**			
Avasimibe	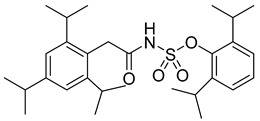	EC50 296 pg/µL	[152]
Daclatasvir	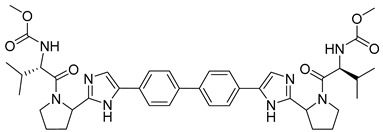	EC50 9−146 pM	[158]
6-(4-chloro-2-methylphenoxy)pyridin-3-amine	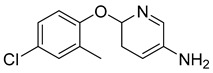	EC50 80 nM	[161]
Fluoxazolevir	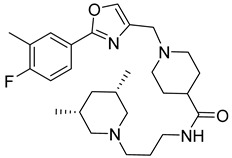	EC50 1.19 µM	[163]
Chlorcyclizine hydrochloride	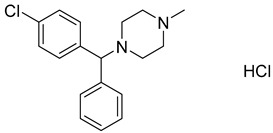	EC50 44 ± 11 nM	[164]
SL201	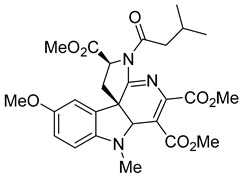	EC50 8.1−8.8 µM	[178]
Molecule 2	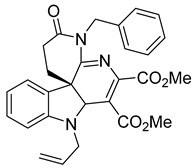	EC50 2.3−3.2 µM	[169]
**Dengue virus**			
ST-148	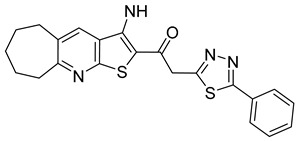	EC50 12−73 nM	[174]
VGTI-A3	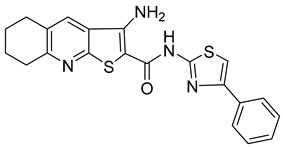	IC50 0.11 µM	[177]
VGTI-A3-03	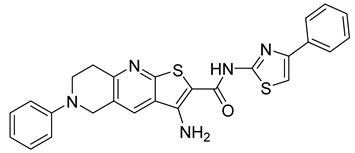	IC50 25 nM	[177]

## 6. Coronavirus Assembly Inhibitors

The coronaviruses are enveloped RNA viruses that assemble and bud at the lumen of the ER-Golgi intermediate compartments and then exit the cell by exocytosis. The key players mediating the assembly of coronaviruses are the structural membrane (M) and envelope (E) proteins (for review see [179]). They provide the signal sequence for accumulation in the ER and formation of the ribonucleoprotein that includes the N (nucleocapsid) protein, which is responsible for specific packaging of genomic RNA through its N-terminal RNA binding domain. The three-dimensional structure of the M protein is still missing and the only available structure is the ab initio model generated by Mahtarin et al. [180]. In contrast, the crystal structures of the N- and C-terminal folded domains of the SARS-CoV-2 N protein have been recently solved [181,182,183]. The NMR structure of the transmembrane pore-forming homopentameric cation channel of E protein has been published as well [184]. This opens a way to rationally design inhibitors of either RNA binding or N-protein oligomerization.

Based on molecular docking, several compounds have been proposed to inhibit the interactions mediated by M, E, and N proteins [185]. Interestingly, they fall into the groups of naturally occurring bioflavonoids or widely manufactured antibacterial and antiviral drugs. E protein was bound by rutin and doxycycline, whilst protease inhibitors simeprevir and grazoprevir (Table 4) showed in silico high affinity to the N protein. The M protein readily interacted with rutin and caffeic and ferulic acids. All identified inhibitors exhibited satisfactory oral bioavailability according to the Lipinski’s rule of five [186]. Multiple computational approaches focused on docking several antivirals into the C-terminal domain of the N protein showed the highest affinity of eukaryotic translation initiation factors inhibitor 4E1RCat (4-[(3E)-3-[[5-(4-nitrophenyl)furan-2-yl]methylidene]-2-oxo-5-phenylpyrrol-1-yl]benzoic acid)(Table 4), followed by rapamycin (Table 4), silmitasertib, TMCB (inhibitor of casein kinase II), and sapanisertib [187]. The rapamycin repurposing in SARS-CoV-2 infection has been recently reviewed [188,189].

Another docking-based approach focused on SARS-CoV-2 assembly inhibitors resulted in the identification of colchicine and some of its derivatives as the top binders of the M protein [190]. Fluorescence HTS assay was designed to screen microarray libraries of compounds immobilized onto isocyanate-coated glass slides by monitoring their binding to the N protein labeled with fluorescent dye Cy5 [191]. The screening identified cephalosporine-derived β-lactam antibiotic ceftriaxone as the best ligand binding both the N-NTD and N-CTD, whilst related antibiotics, cefuroxime and cefotaxime, bound readily to the receptor binding domain of the viral spike protein.

**Table 4:** List of coronavirus assembly inhibitors.

Compound	Structure	Inhibition Efficiency	Ref.
Simeprevir	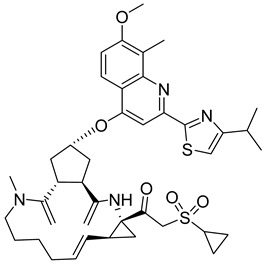	EC50 4.08 µM	[192]
Grazoprevir	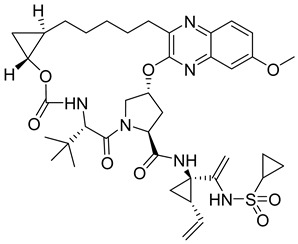	EC50 42 µM	[193]
4E1RCat	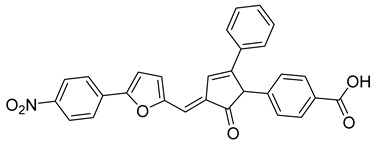	N.D.	[194]
Rapamycin	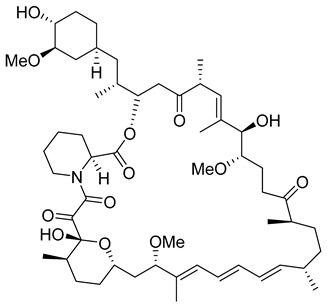	N.D.	

## 7. Picornavirus Assembly Inhibitors

Picornaviruses are small non-enveloped RNA viruses that cause many mild diseases, including common rhinitis. However, in some cases they cause serious, even life-threatening or paralytic diseases, such as poliomyelitis, encephalitis, meningitis, diarrhea, or myocarditis [195]. Effective vaccines are available for some of them, but there are no approved vaccines or drugs on the market for others. Many developed antivirals against picornaviruses failed FDA approval because they usually had low efficiency and many side effects. Thus, the search for new inhibitors of these viruses is still highly desirable.

Picornaviridae include several genera, such as clinically very important genus Enterovirus comprising species enterovirus, rhinovirus, coxsackievirus, echovirus, and human poliovirus and genus Hepatovirus, including species hepatovirus. Unfortunately, they differ in their life cycles on the structure-functional basis, therefore the design of new antivirals is complicated, and often affects only some of these viruses, and thus research and subsequent virus inhibitory tests need to be targeted on each genus and in some cases even on each species separately.

The capsid of picornaviruses is made of four structural capsid proteins, VP1-VP4. It contains a small hydrophobic pocket, formed mostly by VP1 protein, which is necessary for the attachment of the virus to the host cell. Thus, compounds that bind to this pocket, displacing the natural viral pocket-binding factor, were shown to efficiently block the virus life cycle at the virus entry step. Some of these so-called “capsid-binders” were shown to be very effective, with IC50 or EC50 in nM to even pM ranges [196,197,198,199,200]. Unfortunately, the geometry of the binding pockets varies among the rhinoviruses, which prevents the design of “capsid binders” generally targeting rhinoviruses.

Among the most studied molecules that bind to the VP1 pocket are cyclic organic compounds, mostly isoxazole derivatives. The binding of these molecules leads to stabilizing virus protein shell, influencing structural changes necessary for the capsid binding to its host cell receptor, and further blocking subsequent virus uncoating [201,202,203].

The development of antipicornaviral compounds from the group of VP1 capsid pocket binders has begun about 40 years ago by the synthesis of various chemicals firstly based on beta-diketones [204,205,206,207] by the Sterling-Winthrop company. These molecules were named WIN compounds. After several modifications compound arildone (WIN 38020) (Table 5) was discovered and tested [208,209]. Further research and developments of structurally similar molecules resulted in syntheses of tens or even hundreds of molecules with antipicornaviral activity, based on isoxazole (Table 5).

The study of isoxazole and its derivatives (Table 5) led to the synthesis of various substitutes for example disoxaril, 5-[7-[4-(4,5-dihydro-2-oxazolyl)phenoxy]heptyl]-3-methylisoxazole (WIN 51711) [197,198,210,211], its derivative WIN 54954 [212] or WIN 52084 [213] and finally leading to the currently well-known and highly studied antipicornaviral compound pleconaril (WIN 63843), [214], which has also entered clinical trials [215]. Pleconaril was further developed and new derivatives were synthesized and tested for their activities, for example by Shia et al. [216]. Computer-aided drug design has also been used to predict new molecules using pleconaril scaffolding. It led to new sets of highly active pyridylimidazolidinone molecules (PR66) (Table 5). Chang et al. [217] synthesized and tested by in vitro neutralization test a series of pyridylimidazolidinone derivatives, namely 1-[5-(4-arylphenoxy)alkyl]-3-pyridine-4-ylimidazolidin-2-one derivatives (Table 5) that could effectively bind to CA of enterovirus EV71. Some of them were further modified and their antiviral effect was highly improved, with IC50 values in lower nanomolar concentrations. Due to such high activity together with low cytotoxicity, they belong to promising antivirals. Their inhibitory effect was successfully tested also on several other picornaviruses (echovirus, rhinovirus, coxsackievirus, and enterovirus EV38) [217].

Many sets of other molecules based on modified (oxazolinylphenyl)isoxazoles have been synthesized and tested against human rhinoviruses [218]. Skeletons of WIN compounds were also used as templates for computer-assisted drug design to identify new imidazolidinone derivatives with significant antiviral activity against enterovirus EV71. Compounds 1 and 8 (Table 5) were thus identified as potent antivirals not only against EV71 but also the coxsackie virus and they also showed some moderate activity against EV38 and echovirus [216].

Another structure, based on phenoxylimidazol salt, was developed by Schering-Plough and named SCH38057 (Table 5). It was found that it inhibits several entero and rhinoviruses with micromolar EC50 [219]. Its further development led to the synthesis of the compound pocapavir, which also inhibited the broad-spectrum of picornaviruses, but unfortunately, it was not active against rhinoviruses. When resistant strains emerged during the treatment with this drug, it was no longer tested and did not enter clinical trials [220].

Except for pleconaril, other currently studied capsid binders that have entered clinical trials include disoxaril, pirodavir, pocapavir, and vapendavir [220,221,222,223,224] (Table 5).

Vapendavir development started also about 30 years ago at the Janssen Research Center. Compound 3-methoxy-6-[4-(3-methylphenyl)-1-piperazinyl]pyridazine, named R61837 (Table 5), was proved to inhibit many rhinovirus serotypes [225]. Further modifications led to the synthesis of compound pirodavir and its further development to increase its rather low bioavailability, resulting in the synthesis of vapendavir. Vapendavir was shown to be the most potent analogue amongst the benzoxazole and benzothiazole derivatives of pirodavir, even more potent than pleconaril. Vapendavir successfully passed Phase I and part of phase II clinical trials, but then these tests were discontinued [226]. Also, other promising molecules from this series showed undesirable side effects and insufficient efficacy in clinical trials, and except pleconaril, none made it to market. The clinical use of pleconaril has been limited to its use only in the case of life-threatening enterovirus infections [227,228].

Besides antiviral effects, some authors focused on detailed mechanism of action of newly synthesized antiviral compounds on capsid protein and virus life-cycle, as was shown for isoxazole-based compound, WIN 51711, in the study of Plevka et al. [229]. The authors solved the high-resolution structure of enterovirus EV71 in complex with WIN51711 and based on the observed results they confirmed the previously predicted mechanism of its action by stabilizing the viral capsid and thus influencing the capsid dynamics necessary for the virus genome release.

Structure-based rational design of VP1 hydrophobic pocket was also performed to design new capsid pocket binders. Two very promising compounds, named ALD and NLD (Table 5), were found and it was shown that they are very potent against enterovirus EV71, with inhibitory concentration IC50 in the nano- to the picomolar range [230].

A class of about 80 pyrazolopyrimidines compounds has been shown to be quite promising due to their strong and broad spectrum of antiviral activities. In particular, the compound 3-(4-trifluoromethylphenyl)amino-6-phenylpyrazolo[3,4-d]pyrimidine-4-amine appeared to be a promising drug candidate. Another recently discovered molecule, pyrazolopyrimidine named OBR-5-340 (unpublished structure) was shown to act similarly to other capsid pocket binding inhibitors. However, by using cryo-EM, it was shown to bind to a slightly different site, near the pocket, but with a different binding geometry than most other capsid binders [231]. This molecule was active also against pleconaril-resistant picornaviruses.

A novel recently discovered inhibitor of enterovirus EV71 (named G197) (Table 5) is based on a structural chimera of vapendavir and previously discovered EV71 inhibitor, pyridyl imidazolidinone compound BPROZ-194 (Table 5) [232]. It has higher antiviral activity than vapendavir alone, in addition to low cytotoxicity. The authors tested G197 also in a combination with an inhibitor targeting the host phosphatidylinositol 4-kinase III beta, an enzyme necessary for viral replication. This combination drug represents another promising antiviral approach that deserves further development for clinical tests [233].

Benzothiophenes, their derivatives and analogues represent another recently discovered group of antivirals by Kim et al. [234]. It was shown that some of them share the similar capsid-binding mode as pleconaril.

Except for the capsid VP1 hydrophobic pocket, two more capsid regions could be targeted by antivirals. One of them is the region of positively charged amino acids surrounding the capsid five-fold axis, including VP1. This region is essential for virus-host cell binding [235,236]. Of the compounds targeting this region, most important are suramin [237,238], its derivative NF449 (Table 5), tryptophan dendrimers that inhibit also HIV-1 [239], heparan sulfate mimetics [240,241], sulfonated food azo dye E151 (Table 5) [242], or rosmarinic acid (Table 5) [243].

The last capsid region that could be effectively targeted by antivirals is an interprotomer region harboring VP1-VP3. 4-dimethyl amino benzoic acid and its analogues were synthesized and were shown to efficiently bind to this region, thus inhibiting for example coxsackie virus B3 replication. By molecular modeling, a small pocket in VP was shown to be the interacting place [244]. Another molecule, benzenesulfonamide derivative, compound 17 (Table 5) [245], is active against several picornaviral species. Cryo-EM analysis showed that this molecule binds to the pocket between two units of VP1 and one VP3 at the interface of the interprotomer [245].

Another strategy of how to efficiently inhibit picornaviral life cycle and infection is to use monoclonal antibodies targeted to their capsid. Bostina [246] tested this strategy against the hepatitis A virus and by the use of cryo-electron microscopy he also characterized a well-conserved antigenic site of the picornaviral capsid that is recognized by various monoclonal antibodies.

The major drawback for the use of various direct-acting antivirals that target picornaviral capsid is the rapid emergence of resistant mutants [247]. It was shown that even single point mutation in the abovementioned capsid VP1 pocket can lead to virus resistance [248]. Thus, most likely a combination of several antivirals targeting different sites in the virus capsid or even different steps of the virus life cycle could be an effective strategy to prevent failure of the treatment.

**Table 5:** List of compounds targeting capsid protein or capsid of picornaviruses.

Compound	Structure			Inhibition Efficiency	Ref.
WIN 38020 (Arildone)	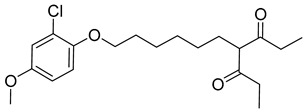			IC50 8.9 μM MIC 0.2 μM	[209]
WIN 51711 (Disoxaril)	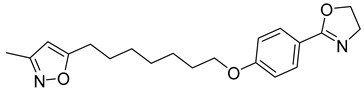			EC90 0.03−0.3 µg/ml	[197]
WIN 54954	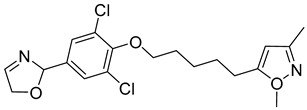			EC80 0.28 µg/ml	[249]
WIN 52084	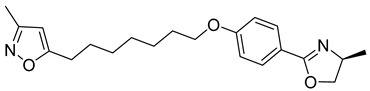			N.D.	
WIN 63843 (Pleconaril)	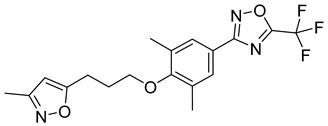			EC50 0.13−0.44 µM	[250]
PR66	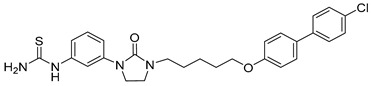			N.D.	
1-[5-(4-Arylphenoxy)alkyl]-3-pyridin-4-ylimidazolidin-2-one derivatives	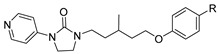	14a	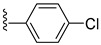	IC50 9.3 nM	[217]
		28b	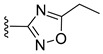	IC50 0.5 nM	[217]
		32b	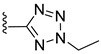	IC50 0.9 nM	[217]
SCH38057	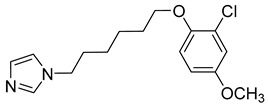			EC50 10.2−29.1 μM	[219]
Pirodavir	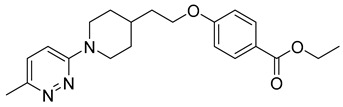			EC50 0.14−33 µM	[250]
Pocapavir	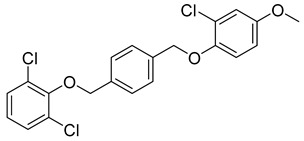			N.D.	
Vapendavir	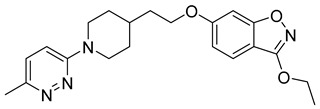			EC50 0.04−2.6 µM	[250]
NLD	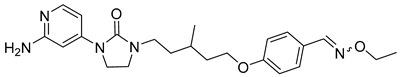			IC50 25 pM	[230]
ALD	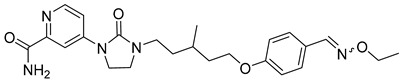			IC50 8.54 nM	[230]
R61837	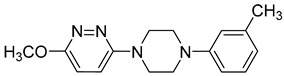			MIC 18 nM	[225]
G197	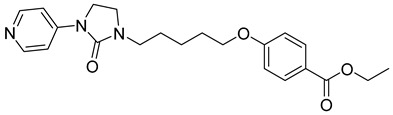			N.D:	
BPR0Z-194	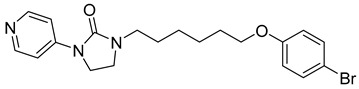			IC50 0.1−10.34 µM	[232]
NF449	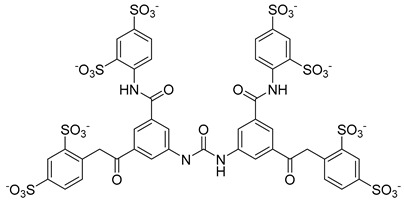			IC50 4 μM	[238]
E151	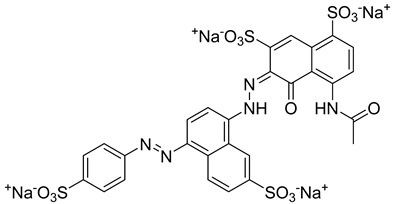			IC50 2.39−28.12 μM	[242]
Rosmarinic acid	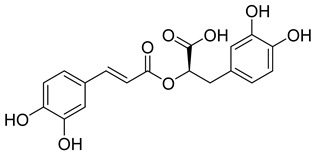			N.D.	
Compound **17**	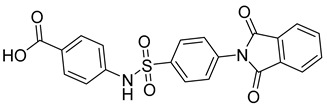			EC50 0.7 ± 0.1 μM	[245]

## 8. Conclusions

Many various antivirals that are currently available on the market exhibit unpleasant or even harmful side effects. In addition, many antivirals, when used for prolonged periods, cause viruses become resistant. Therefore, it is very important to constantly search for new inhibitors that target different steps in the viral life cycle. Here, we have summarized current information about the most relevant compounds that target capsid proteins or viral capsids of clinically relevant virus genera with RNA genomes or using RNA intermediate in their life cycle.

The search for effective antivirals targeting either the viral capsid protein or the assembled capsid was driven by the efforts to obtain inhibitors with a different mechanism of action than commonly used viral enzyme inhibitors and to avoid the risk of resistant mutants through combination therapy, or to have an alternative therapeutic molecule in case of already developed resistance. These compounds can be divided into several groups: inhibitors of the virus capsid assembly, or disassembly, inhibitors preventing the binding of the virus capsid to the host cell receptor, or inhibitors that via their interaction with the capsid protein block packaging of the viral genome. Besides routine applications of the inhibitors, some new strategies have been developed to specifically target the inhibitor to the virus. These involve the fusion of capsid proteins with antiviral molecules that are co-assembled into a virus particle. Antibodies to viral capsid proteins can also be used as potent antivirals. Numerous approaches, including high-throughput screening of available libraries of chemical compounds, targeted drug design, in silico modelling, chemical modifications of currently known compounds, or by the repurposing of already approved drugs have led to the discovery of new inhibitors either of biological or synthetic origin. Their structures range from small organic compounds binding to hydrophobic or interprotomer pockets of the viral capsid, through selected or randomly combined peptides or their fragments that bind to capsid protein interaction interfaces, to compounds binding to capsid protein domains important for their interactions with other viral proteins, genomic nucleic acids, host proteins, or factors necessary for the virus life cycle. The search for inhibitors has helped to elucidate the capsid protein-driven steps of the virus life cycle. Despite some remaining challenges, significant progress has been recently made in the development of effective capsid-targeted inhibitors which, due to their interaction with the essential and thus conservative domains of capsid proteins, represent a very promising alternative to drugs in combination or single-compound therapies.
